# CD8^+^NKT细胞表面活化性受体NKG2D在肺癌患者外周血中的表达及临床意义

**DOI:** 10.3779/j.issn.1009-3419.2010.10.06

**Published:** 2010-10-20

**Authors:** 妮 程, 福才 韩, 艳峰 王, 羡霞 麦, 文 苏

**Affiliations:** 1 030001 太原，山西医科大学第二临床医学院 Second Clinical Medical College, Shanxi Medical University, Taiyuan 030001, China; 2 030001 太原，山西省肿瘤医院呼吸内科 Department of Respiratory Medicine, Shanxi Provincial Tumor Hospital, Taiyuan 030001, China; 3 030001 太原，山西省肿 瘤医院研究所免疫室 Laboratory of Immunology Research Institute, Shanxi Provincial Tumor Hospital, Taiyuan 030001, China

**Keywords:** CD8^+^NKT, NKG2D, 肺肿瘤, 免疫逃逸, CD8^+^ Natural killer T cell, NKG2D, Lung neoplasms, Immune escape

## Abstract

**背景与目的:**

NKT细胞活化性受体NKG2D及sMICA是近来肿瘤免疫研究领域的热点之一。本研究旨在观察肺癌患者外周血中CD8^+^NKT细胞受体NKG2D表达水平的变化，并对NKG2D及sMICA进行相关性分析，探讨它们在肺癌免疫监视中的作用及临床意义。

**方法:**

选择82例初发未治疗的肺癌患者，采用流式细胞术检测外周血CD8^+^NKT细胞活化性受体NKG2D的表达，并以45例健康人作对照，采用酶联免疫吸附法检测肺癌患者血清中sMICA的表达，分析NKG2D与肺癌临床生物学特征的关系。

**结果:**

肺癌患者外周血中CD8^+^NKT细胞表面活化性受体NKG2D水平均低于对照组，差异有统计学意义（*P* < 0.001）。随TNM分期的增加，NKG2D的表达率逐渐降低。其中Ⅳ期肺癌患者NKG2D的表达明显低于Ⅰ期-Ⅱ期及Ⅲ期患者该受体的表达，差异有统计学意义（*P* < 0.001）。肺Ca患者中吸烟人群外周血中CD8^+^NKT细胞受体NKG2D的表达较非吸烟者低，差异有统计学意义（*P* < 0.05）。CD8^+^NKT细胞受体NKG2D与sMICA呈负相关（*r*=-0.598, *P* < 0.001）。

**结论:**

肺癌患者CD8^+^NKT细胞表面受体NKG2D在外周血中低表达，且与肺癌的分期有关；该受体的下调通过血清中sMICA上调的机制参与了肺癌以肿瘤为中心抑制免疫网络的形成；监测NKG2D及sMICA有助于了解患者的免疫功能，可为临床肿瘤的综合治疗提供依据。

## Abstract (en)

**Background and objective:**

NKG2D-expressing CD8^+^NKT cells and soluble major histocompatibility complex class Ⅰ-related chain A (sMICA) is one of recently emerged general interests in tumor research area. The aim of this study is to investigate the levels of NKG2D-expressing CD8^+^NKT cells in peripheral blood of lung cancer patients, which are remarkably related to clinical significance, and to analyze the correlation between NKG2D-expressing CD8^+^NKT cells and sMICA levels, and to explore the role of NKG2D and sMICA in immune surveillance.

**Methods:**

Flow cytometry was used to determine the percentage of NKG2D-expressing CD8^+^NKT cells, and ELISA was used to measure the levels of sMICA in peripheral blood of 82 untreated lung cancer patients and 45 healthy controls. The association of NKG2D levels with clinical features was analyzed.

**Results:**

The expression of NKG2D on CD8^+^NKT cells in lung cancer group was significantly lower than that in healthy group, with statistically significant difference (*P* < 0.001). And with the increase of TNM stage, NKG2D expression rate reduced gradually. NKG2D expression in stage Ⅳ disease was significantly lower than which stage Ⅰ-Ⅱ and Ⅲ disease (*P* < 0.001). The expression of NKG2D on CD8^+^NKT cells was remarkably lower in that in smokers than that in non-smokers, with statistically significant difference (*P* < 0.05). NKG2D exhibited negative correlation with sMICA (*r*=-0.598, *P* < 0.001).

**Conclusions:**

Lung cancer has low expression of NKG2D in CD8^+^NKT cells which correlate with pathological stage. Detection of NKG2D and sMICA might be helpful to understand immune functions and provide evaluation of combined treatment for clinical cancer.

肿瘤免疫编辑学说^[1, 2]^认为机体的免疫系统和肿瘤细胞之间存在着相互作用：免疫系统重塑肿瘤细胞的抗原性；肿瘤细胞改变免疫效应细胞的抗肿瘤效应。其中在抗肿瘤免疫中，活化性受体NKG2D为近几年来研究的热点之一。它在所有的NK细胞、大多数NKT细胞、巨噬细胞和γδ^+^T细胞表面均有表达，另外NKG2D也存在于CD8^+^T细胞表面^[3]^。其配体之一是主要组织相容性复合体Ⅰ类相关分子A（major histocompatibility complex class Ⅰ chainrelated A, MICA）。靶细胞表面NKG2D的配体与淋巴细胞表面NKG2D的结合可以激活淋巴细胞杀伤表达NKG2D配体的肿瘤细胞^[4]^。因此，NKG2D在肿瘤免疫中发挥重要作用。CD3^+^CD56^+^NKT细胞作为一类新的细胞毒性效应细胞，兼有T淋巴细胞强大的抗瘤活性和NK细胞的非MHC限制性广谱杀瘤作用。但是人们对活化受体NKG2D的认识仅局限于NK细胞及T细胞，该受体在NKT细胞表面的表达情况及其与肺癌临床生物学关系尚不明了。本研究旨在对CD8^+^NKT细胞表面活化性受体NKG2D在肺癌患者外周血中的表达水平及其临床意义进行初步探索。

## 1 材料与方法

### 1.1 材料

#### 1.1.1 研究对象

实验组选取2009年11月-2010年5月山西省肿瘤医院呼吸内科收治的初治肺癌患者82例，年龄43岁-69岁，中位年龄54岁。入选标准为具有完整临床、病理资料、经病理或细胞学、影像学检查确诊的肺癌患者，所有患者均无免疫相关性疾病和其它肿瘤病史，且无使用激素类药物及免疫抑制剂史。根据UICC国际TNM标准2009分类，Ⅰa期-Ⅱb期肺癌患者22例，Ⅲa期-Ⅲb期肺癌患者28例，Ⅳ期肺癌患者32例。根据病理分型分类，鳞癌45例，腺癌37例（不研究小细胞癌）。对照组来自体检职工部分志愿者45例，其中男25例，女20例，年龄40岁-67岁，中位年龄52岁。

#### 1.1.2 实验试剂与仪器

抗CD3-PerCP、抗CD8-PE、抗CD56-FITC、抗NKG2D-APC四种荧光素直接标记的抗体购自美国BD公司；sMICA酶联免疫吸附实验（ELISA）试剂盒为德国IMMATRICS Biotechnologies产品；裂解液购自美国Becton Dickinson公司；PBS磷酸盐缓冲液（pH=7.2-7.4）；Sunrise全自动酶标仪购自奥地利Tacan公司；FACSAria流式细胞仪购自美国BD公司；KDC-1044低速离心机购自科大创新股份有限公司中佳分公司。

### 1.2 方法

采用四色免疫荧光染色（CD3-PerCP/CD8PE/CD56-FITC/NKG2D-APC）方法检测受体NKG2D在CD8^+^NKT细胞表面的表达水平。治疗前抽取患者空腹静脉血2 mL于EDTA钾盐抗凝的真空采血管中，轻轻摇匀后备用。取荧光标记的单克隆抗体20 μL加入EP管中，设相应的同型对照，加入EDTA钾盐抗凝全血100 μL，充分混匀，室温避光孵育20 min；同时加入1 mL红细胞裂解液，混匀后避光孵育10 min，离心7 min（1 500 rpm），弃去上清；加入2 mL PBS洗涤液后离心7 min（1 500 rpm），弃去上清，重复2次，弃去上清PBS，将洗涤液混匀，流式细胞仪上机检测，采用Diva软件分析获取细胞。sMICA根据试剂盒提供的操作方法测定，以波长450 nm检测结果。测定sMICA水平的样本获取：取静脉血2 mL-3 mL于无抗凝真空采血管中，待血液凝固后，2 000 rpm，离心10 min，取血清于1.5 mL的EP管中，-20 ℃保存备用。

### 1.3 统计学方法

采用SPSS 13.0统计软件进行。结果用Mean±SD表示。各实验组之间表达水平差异采用独立样本*t*检验或*One-Way ANOVA*进行分析；双变量相关性分析根据变量是否符合正态分布采用*Pearson*相关分析或*Spearman*相关分析。以*P* < 0.05为有统计学差异。

## 2 结果

### 2.1 对照组健康人群与肺癌患者外周血CD8^+^NKT细胞受体NKG2D表达情况

与对照组健康人群相比，患者外周血中CD8^+^NKT细胞受体NKG2D的表达下降（*P* < 0.001）（表 1，图 1）。

**1:**
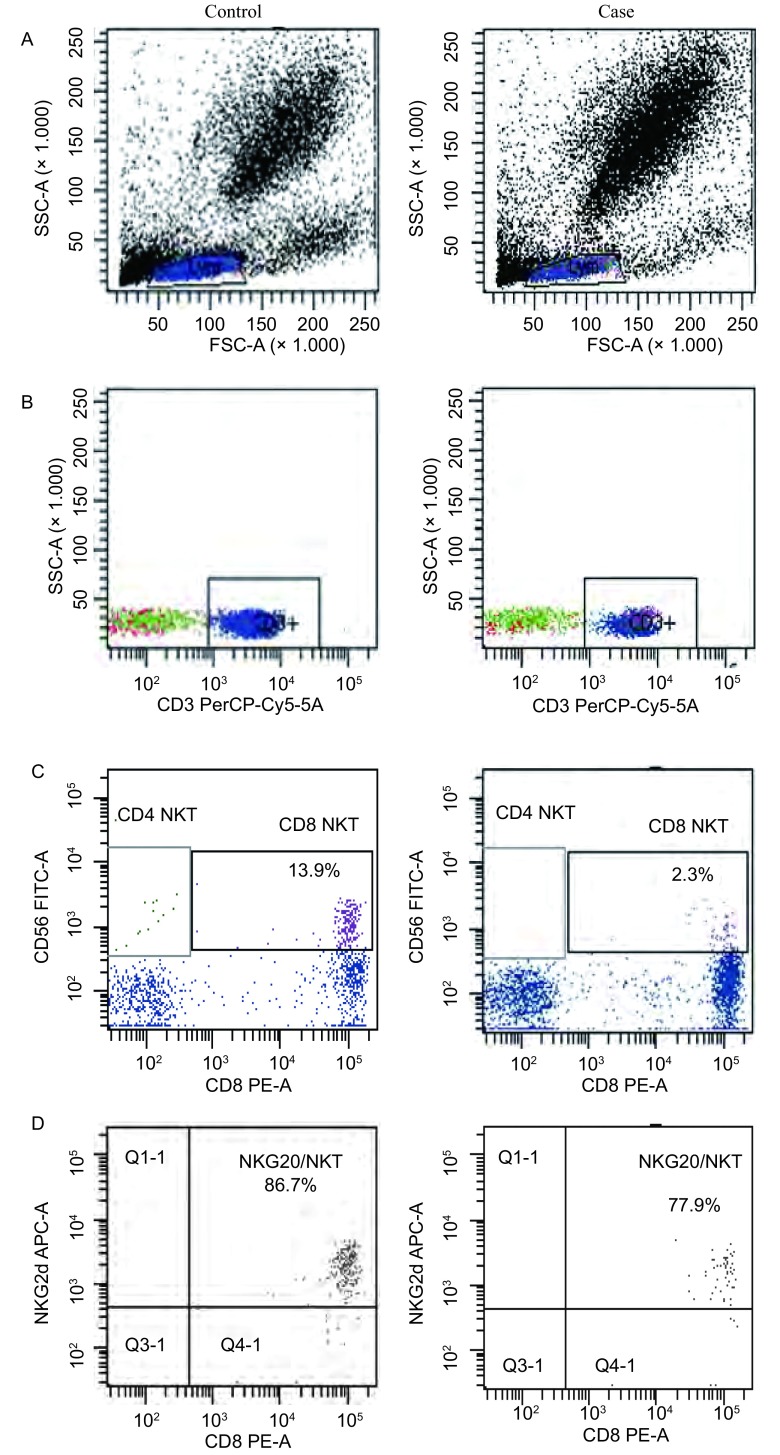
对照组健康人和肺癌患者CD8^+^NKT细胞受体NKG2D流式细胞散点图 Scatter plots about NKG2D-expressing CD8^+^NKT cells which was measured by flow cytometry for healthy controls and lung cancer patients

**1:** 正常人和肺癌患者外周血CD8^+^NKT细胞受体NKG2D表达水平 NKG2D expression levels in CD8^+^ NKT cell of normal subjects and lung cancer patients

Group	*n*	Expression of NKG2D		
		（Mean±SD）%	*t*	*P*
Control	45	83.52±4.95	-7.77	< 0.001
Lung cancer patient	82	75.73±5.64		

### 2.2 肺癌患者外周血CD8^+^NKT细胞受体NKG2D与生物学

特征之间的关系与非吸烟患者相比，吸烟患者外周血中CD8^+^NKT细胞受体NKG2D的表达下降（*P* < 0.05）。CD8^+^NKT细胞受体NKG2D的表达与病理类型、性别、年龄无关（*P* > 0.05）。随着TNM分期的增加，NKG2D的表达率逐渐降低。其中Ⅳ期肺癌患者NKG2D的表达低于Ⅰ期-Ⅱ期及Ⅲ期患者该受体的表达，差异有统计学意义（*P* < 0.001）（表 2）。

**2:** 肺癌患者外周血CD8^+^NKT细胞受体NKG2D表达与一般生物学特征的关系 Relationship between the expression of NKG2D-expressing CD8^+^NKT cells and biological characteristics of advanced lung cancer patients

Characteristic		*n*	NKG2D		
			(Mean±SD)%	*t*	*P*
Gender				-1.669	0.099
	Male	48	77.99±5.53		
	Female	34	80.20±6.45		
Age				1.457	0.215
	≤50	24	81.64±7.23		
	51-60	32	77.43±5.66		
	≥61	26	78.12±6.32		
Smoking istory				6.254	< 0.05
	Smoker	49	75.06±4.84		
	Non-smoker	33	82.39±6.14		
Clinical stage				13.584	< 0.001
	Ⅰ-Ⅱ	22	83.51±6.80		
	Ⅲa-Ⅲb	28	80.62±7.16		
	Ⅳ	32	74.67±5.41		
Histological type				1.46	0.825
	Squamous cell carcinoma	45	81.23±5.14		
	Adenocarcinoma	37	79.82±6.37		

### 2.3 外周血CD8^+^NKT细胞受体NKG2D与sMICA的相关性

经线性相关分析表明，外周血CD8^+^NKT细胞受体NKG2D和sMICA呈线性负相关（*r*=-0.598, *P* < 0.001）（图 2）。

**2:**
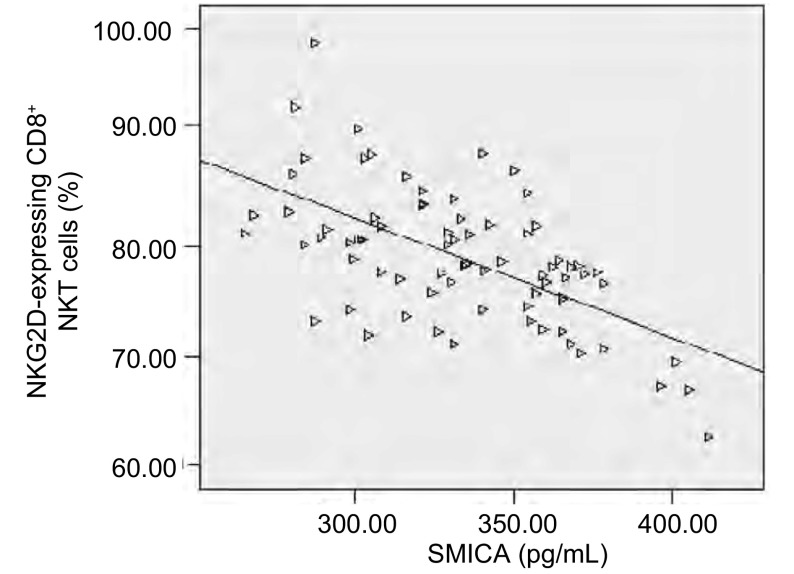
外周血CD8^+^NKT细胞表面受体NKG2D与SMICA负性相关散点图 Scetter Plots about sMICA levers and the number of NKG2D-expressing CD8^+^NKT cells which are negatively corrected

## 3 讨论

肺癌是最常见的恶性肿瘤之一，其发生发展与机体的免疫状态尤其是细胞免疫密切相关。因此，随着多学科治疗模式的逐步形成，免疫治疗作为继手术、化疗及放疗后第四种肿瘤治疗手段受到人们的广泛重视。自然杀伤性T细胞（natural killer T cell, NKT）是一类在机体早期抗肿瘤免疫机制中起重要作用的效应T细胞，作为机体抗肿瘤的第一道防线，其在抵抗肿瘤和病毒感染过程中起关键作用。NKT细胞以CD3^+^CD56^+^为生物学特征。根据CD4、CD8的表达与否，NKT细胞分为CD4^+^NKT、CD8^+^NKT和（CD4-CD8-）DN三个亚群^[5]^。NKT细胞通过识别由非经典的MHC-Ⅰ类分子（CD1d）递呈的糖脂类抗原活化并迅速分泌大量TH1和TH2类细胞因子^[6, 7]^。NKG2D是自然杀伤性T细胞重要的激活性受体，它通过识别靶细胞（多为肿瘤细胞）表面的配体MICA，并与之结合传递活化信号，继而激活免疫效应细胞（包括NKT细胞）对靶细胞的杀伤作用。因此NKG2D的表达是机体抗肿瘤免疫的关键因素。sMICA是由于肿瘤细胞的死亡、MICA分泌和金属蛋白酶水解而脱落至外周血中的MICA。sMICA降低了肿瘤表面MICA分子的表达水平，而且它还能诱导NKT细胞表面和NK细胞表面受体NKG2D发生内化降解，导致NKT细胞和NK细胞NKG2D的下调，抑制免疫细胞杀伤活性，导致肿瘤逃逸^[8]^。

CD8^+^NKT细胞表面表达NKG2D的同时，NK细胞也表达CD56，因此本研究对NKG2D表达的流式细胞结果进行分析时，选择CD3^+^CD56^+^的细胞群，有效避免了NK细胞（CD3-CD56^+^）的干扰，确保了实验结果的准确性。本研究结果表明NKG2D发挥免疫正调节作用。Cristina等^[9]^研究发现黑色素瘤患者及结直肠癌患者免疫细胞受肿瘤细胞的编辑，其细胞表面的活化性受体NKG2D表达下降时，NKT细胞的杀伤活性降低；CD8^+^NKT细胞表面受体NKG2D的表达明显低于健康对照组，同时其配体sMICA高于健康对照组；外周血sMICA上调介导的NKT细胞活化受体NKG2D的下调机制参与了以肿瘤为中心抑制免疫网络的形成，与机体免疫状态有着密切联系，可作为监视肿瘤患者免疫功能的参考指标，也可作为评估肿瘤发生发展的参考依据。

肺癌患者细胞免疫处于抑制状态，而机体的免疫状态与肿瘤的侵袭及演进有关。NKG2D表达的检测可作为评价肺癌细胞免疫状态的指标，在肺癌免疫监视中发挥重要作用。本研究结果表明，与健康对照组人群相比，肺癌患者外周血中CD8^+^NKT细胞表面NKG2D受体的表达水平较低（*P* < 0.001）。笔者认为肺癌患者外周血NKT细胞表面受体NKG2D下调的原因可能是：①血清高表达sMICA诱导了NKT细胞表面的NKG2D发生内化降解^[10]^；②与肿瘤分泌有关的一些免疫抑制细胞因子如TGF-β有关^[11]^；③可溶性抗原sMICA可作为封闭因子封闭效应NKT细胞表面受体NKG2D，阻止NKT细胞的细胞毒作用，由于其结合位点被占用，因此使用流式细胞仪无法检测到这部分受体的存在，导致所测NKT细胞表面受体NKG2D水平降低。④肺癌细胞表面脱落的sMICA反过来可以下调NKG2D受体的表达而产生肺癌免疫逃逸。

肿瘤的免疫治疗建立在免疫系统的监视功能的基础上，即免疫系统能够识别肿瘤细胞并将其杀死。本研究结果表明，肺癌患者外周血中CD8^+^NKT细胞表面NKG2D受体的表达较健康人群低（*P* < 0.001），同时该受体的表达与吸烟、临床分期相关（*P* < 0.05），且随着临床分期的增加，NKG2D的表达逐渐降低。其中Ⅰ期-Ⅱ期及Ⅲ期肺癌患者NKG2D的表达均明显高于Ⅳ期患者该受体的表达，差异有统计学意义（*P* < 0.001），但它与肺癌病理分型、年龄及性别无关（*P* > 0.05），说明外周血中CD8^+^NKT细胞表面NKG2D受体与肺癌的发生发展及预后密切相关。研究同时表明，外周血CD8^+^NKT细胞受体NKG2D和其配体sMICA呈线性负相关，间接证实肺癌患者血清sMICA升高介导NKG2D下调的肿瘤免疫逃逸机制，而其中NKG2D发挥免疫正调节作用。综上所述可能由于其表面NKG2D受体表达的下降，肺癌患者体内NKT细胞对肺癌细胞的反应性降低，因此不能对肺癌细胞进行有效地杀伤，同时表面MICA/B配体的表达而逃逸NKT细胞的细胞毒作用。肺癌细胞也可能通过减少其上调介导NKT细胞活化受体NKG2D下调机制参与了晚期肺癌以肿瘤为中心抑制免疫网络的形成，与机体免疫状态有着密切联系，可作为监视晚期肺癌患者免疫功能的参考指标，也可作为评估肺癌发生发展的参考依据。但以N KG2D-MICA为基础的免疫监视功能在肺癌患者中如何发挥作用、如何诱导肺癌细胞表面MICA/B的表达、如何减少膜性MIC的脱落以及如何上调NKG2D的表达以恢复NKT细胞的活性值得深入研究。
